# qPrimerDB 2.0: an updated comprehensive gene-specific qPCR primer database for 1172 organisms

**DOI:** 10.1093/nar/gkae684

**Published:** 2024-08-09

**Authors:** Xiaodong Li, Boyu Meng, Zhi Zhang, Lijuan Wei, Wei Chang, Yuhong Wang, Kai Zhang, Tian Li, Kun Lu

**Affiliations:** Integrative Science Center of Germplasm Creation in Western China (CHONGQING) Science City, College of Agronomy and Biotechnology, Southwest University, Beibei, Chongqing 400715, China; Integrative Science Center of Germplasm Creation in Western China (CHONGQING) Science City, College of Agronomy and Biotechnology, Southwest University, Beibei, Chongqing 400715, China; State Key Laboratory of Resource Insects, Southwest University, Chongqing 400715, China; Integrative Science Center of Germplasm Creation in Western China (CHONGQING) Science City, College of Agronomy and Biotechnology, Southwest University, Beibei, Chongqing 400715, China; Integrative Science Center of Germplasm Creation in Western China (CHONGQING) Science City, College of Agronomy and Biotechnology, Southwest University, Beibei, Chongqing 400715, China; Integrative Science Center of Germplasm Creation in Western China (CHONGQING) Science City, College of Agronomy and Biotechnology, Southwest University, Beibei, Chongqing 400715, China; Integrative Science Center of Germplasm Creation in Western China (CHONGQING) Science City, College of Agronomy and Biotechnology, Southwest University, Beibei, Chongqing 400715, China; State Key Laboratory of Resource Insects, Southwest University, Chongqing 400715, China; Integrative Science Center of Germplasm Creation in Western China (CHONGQING) Science City, College of Agronomy and Biotechnology, Southwest University, Beibei, Chongqing 400715, China; Engineering Research Center of South Upland Agriculture, Ministry of Education, Chongqing 400715, China; Academy of Agricultural Sciences, Southwest University, Beibei, Chongqing 400715, China

## Abstract

High-quality primer design is essential for the success of all polymerase chain reaction (PCR)–based experiments. We previously developed a thermodynamics-based gene-specific quantitative PCR (qPCR) primer database for 147 organisms, which has been used extensively in gene expression studies. However, the number of organisms and the imperfection of function in the database limits its potential applications. Here, we improved the functionality of qPrimerDB to create a more comprehensive primer resource. Specifically, we (i) developed an improved primer design tool, qPrimer, building upon the previous qPrimerDB pipeline, to enhance the efficiency and simplicity of genome-scale qPCR primer design; (ii) pre-computed qPCR primer resources from 1 308 genomes of 1172 organisms and (iii) introduced a complete system for identifying, designing, checking, marking, and submitting qPCR primers. qPrimerDB 2.0 is freely available at https://qprimerdb.biodb.org. The qPrimer source code is available at https://github.com/swu1019lab/qPrimer.

## Graphical Abstract

**Graphical Abstract:**
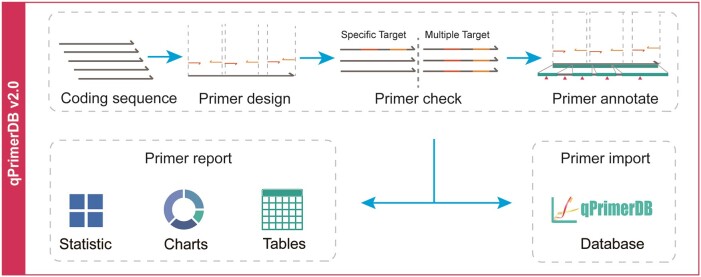

## Introduction

Quantitative polymerase chain reaction (qPCR) is a common technique for detecting and quantifying nucleic acid molecules in biological and environmental samples. This standard technique is used in a wide range of applications including gene expression analysis, RNA interference validation, microarray validation, pathogen detection, genetic testing, and disease research. Optimal primer design is a key parameter affecting the results of PCR experiments. However, qPCR primer design remains a lengthy process in practical scenarios due to filtering constraints (e.g. spanning exon–exon junction, secondary structures, and specificity) or more complex design tasks (e.g. multi-target qPCR).

Recent advances in DNA sequencing techniques have permitted an increasing number of high-quality genome assemblies to be decoded (1–3), providing useful resources for batch primer design. Several databases, such as PrimerBank (4) and MRPrimerW2 (5), containing pre-computed primers have been developed to -facilitate primer design. However, these databases are based on only a few important organisms and are not yet comprehensive enough for broad-scale research applications. qPrimerDB (6) is the most comprehensive qPCR primer database available to date, incorporating large numbers of qPCR primer pairs spanning 147 important organisms. However, the information provided about genes is not uniform, as the whole-genome sequences of these organisms were collected from multiple databases [e.g. Phytozome and Ensembl (7,8)], complicating future updates and management. Additionally, the number of organisms currently in the database is insufficient for satisfying the primer design needs of many experimental biologists.

**Figure 1.**
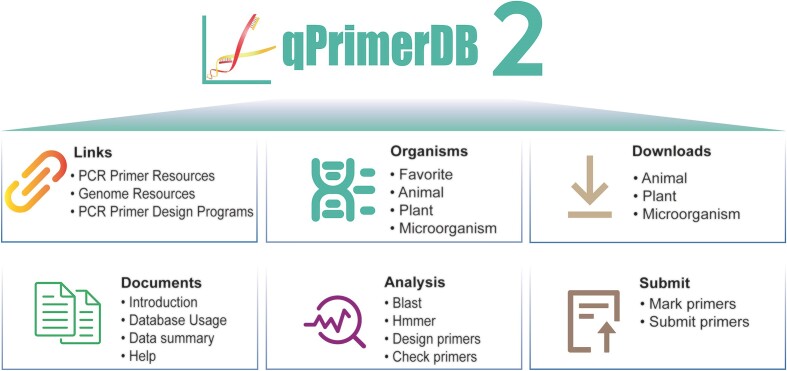
Overview of the qPCR primer Database, qPrimerDB v2.0.

To provide a more comprehensive, uniform platform for researchers and integrate a wide range of sequenced genomes, we made several major updates to the qPrimerDB database. The first involved developing a standard, multi-core primer design tool written in Python for qPrimerDB updates. Second, we downloaded 1 308 genome sequences of more than 1 172 organisms (∼790% increase and ∼697% increase, respectively) from the NCBI Reference Sequence (RefSeq) database (9) and designed 430 million primer pairs for 43 million genes to supplement the updated data. Third, we designed a complete qPCR primer analysis system that offers practical aid for custom primer design. We also redesigned the architecture of the database and website to provide faster, more user-friendly interfaces (Figure 1). Over the past five years, qPrimerDB has been significantly updated regarding data volume and analysis modules (Table 1).

**Table 1.** Statistics of two versions of qPrimerDB

		qPrimerDB 2.0 (2023)	qPrimerDB 1.0 (2018)
Data	Number of species	1172	147
	Number of assemblies	1308	147
	Total number of genes	43 237 052	3 598 514
	Number of genes covered	42 984 629	3 331 426
	Total number of primer pairs	429 822 383	51 091 785
	Percentage of genes covered	99.42%	92.58%
Function	Primer search (RefSeq ID, gene symbol, and other key words)	Available	NA
	Primer design	Available	NA
	Primer check	Available	NA
	Primer mark	Available	NA
	Primer submission	Available	NA
Tool	qPrimer (local running support)	Available	NA

## New features and updates

### Enhanced tool for batch primer design on the genome-wide scale

To identify optimal primer pairs on the genome-wide scale, we introduce qPrimer, an improved qPCR batch primer-design tool based on the Python language. Building on the previous pipeline, qPrimer incorporates a series of new tool including Primer3 (10) and In-Silico PCR (https://genome.ucsc.edu/cgi-bin/hgPcr) to facilitate the design of genome-wide primer sequences for a specific target organism. The widely used Primer3 program for designing PCR primers is written in C code, making it faster for batch primer design. In-Silico PCR uses an indexing strategy to query a sequence database with a pair of PCR primers, delivering fast performance. Locating PCR primers using In-Silico PCR allows users to thoroughly examine the specificity of the primers. Compared to the v1.0 pipeline, we have enhanced batch primer design in the following ways.

#### Standard bioinformatic file formats

Similar to the v1.0 pipeline of qPrimerDB, the qPrimer tool begins with a FASTA-formatted input file containing the coding sequence or transcripts to be searched for primers and allows the user to check their specificity using a reference file such as the whole mRNA or genomic sequence. Additionally, the qPrimer tool can accept a gene annotation file with GTF format to obtain exon spanning information of primers. Considering that single nucleotide polymorphisms (SNPs) present in the template sequence can lead to incorrect primer design, users can now include variation information with BED format in the tool.

#### Improved performance

The updated pipeline's modular design allows users to benefit from a streamlined process (Supplementary Figure S1). To execute the primer design process, users simply need to prepare a suitable input file and call the relevant scripts in the pipeline. Furthermore, qPrimer allows users to specify a broad range of parameters in a configuration file, providing substantially more flexibility over v1.0. Notably, qPrimer supports parallel computing through a central processing unit (CPU) resource, accelerating the primer design process. Compared with the v1.0 pipeline, qPrimer exhibits faster speed and higher gene cover number when testing for Arabidopsis (*Arabidopsis thaliana*), rice (*Oryza sativa*) and maize (*Zea mays*).

#### Abundant parameter constraints

To optimize the binding specificity of the primer pair, we added more parameter constraints. These constraints fall into five main categories, as follows. (i) Penalty value calculations. The numerous constraints that need to be considered when designing primers require a more intuitive measure for evaluating primer quality. Penalty values are computed by factoring in penalty weights for common filter constraints such as melting temperature (*T*_m_), GC percentage, length, and thermodynamic values. The primers are then sorted according to their penalty values and the ones with the lowest penalty are recommended for detecting gene expression. (ii) Thermodynamic secondary structure calculations. Secondary structures arise when primers fold in on themselves or anneal with each other, potentially impacting the yield of high-quality PCR products. Predicting secondary structures can improve primer design by eliminating sequences with a high probability of forming alternative secondary structures. In version 2.0, the propensity for hairpins and dimers to form is assessed using thermodynamic models. (iii) Exon spanning. The number of exons spanned and the genomic position of each primer and amplicon can be determined to select primers spanning an exon–intron boundary; this information can be used to avoid amplifying contaminating genomic DNA. Exon-spanning information will be added as gene annotation information becomes available. (iv) SNP detection. SNPs occurring in primer-binding sites can destabilize oligonucleotide binding and reduce target specificity. To obtain optimal primers, it is imperative to consider whether SNPs are located within a primer sequence. Therefore, the presence of SNPs will be indicated in version 2.0 when variation information is accessible. (v) Specificity checking. Primer end stability is the maximum Gibbs free energy (Δ*G*) value of the last five 3′ bases of a primer, or the maximum Δ*G* value for the binding site. A more stable 3′ end will help reduce false priming. In version 2.0, we choose qPCR primers (previously designated as high-confidence, gene-specific primers) based on the stability of the 3′ end of the primer and requiring a Δ*G* of –9 kcal/mol or greater. Additionally, we designate qPCR primer pairs that align uniquely to the reference sequence as gene-specific primer pairs and recommend them for detecting gene expression.

#### Detailed HTML and JSON report

By default, qPrimer designs ten primer pairs with the lowest penalty for each gene and generates a visual html report file after annotating and specificity checking. In our update, the report file documents the total number of genes, the number of primer pairs per gene, the total number of best and all primer pairs, as well as other information. The entire program's runtime is recorded in a log file. The final primer design results are output in the JSON format, which can be readily interpreted by humans and for further analysis.

### More organisms for batch primer design

**Figure 2.**
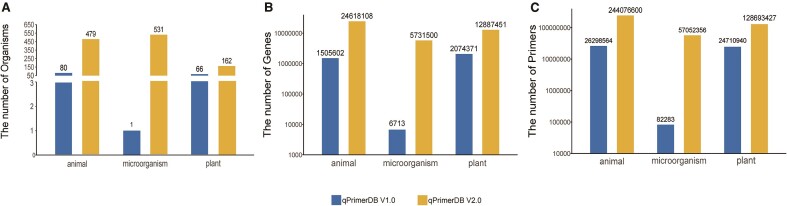
Data volume of two versions of qPrimerDB in animal, plant and microorganism.

Over the past 20 years, reference genomes have been generated for thousands of organisms (1–3). Releasing these reference genomes has dramatically advanced studies in all disciplines of biology and provided valuable resources for batch primer design. The number of organisms and transcripts available from NCBI RefSeq is growing rapidly: 138 491 and 55 021 439 as of 5 September 2023, respectively. In qPrimerDB 2.0, we curated and included a total of 1172 organisms (>160 plants, 500 animals, and 500 microorganisms), 43 million genes, and 430 million primers (Table 2). Compared with v1.0, the number of organisms, genes, and primers is greatly improved (Figure 2). In addition, we included multiple reference-level genomes for major organisms in qPrimerDB 2.0.

**Table 2.** The statistics of qPrimerDB 2.0

	Organisms	Assemblies	Genes	Coverage genes	Primers	Specific target primers	Multiple target primers
Animal	479	553	24 618 101	24 406 571 (99.14%)	244 076 600	73 859 831	170 175 911
Plant	162	202	12 887 451	12 872 191 (99.88%)	128 693 427	64 012 014	64 679 158
Fungi	455	471	4 999 391	4 986 008 (99.73%)	49 858 510	47 669 693	2 188 817
Protist	64	67	698 173	686 824 (98.37%)	6 863 683	5 704 776	1 158 716
Microsporidia	12	14	33 936	33 035 (97.35%)	330 163	313 341	16 822
Total	1172	1307	43 237 052	42 984 629 (99.42%)	429 822 383	191 559 655	238 219 424

### Comprehensive primer analysis system

We implemented a comprehensive online primer analysis system in qPrimerDB 2.0. Furthermore, we propose best practices for selecting qPCR primers, as outlined below.

#### Primer search

**Figure 3.**
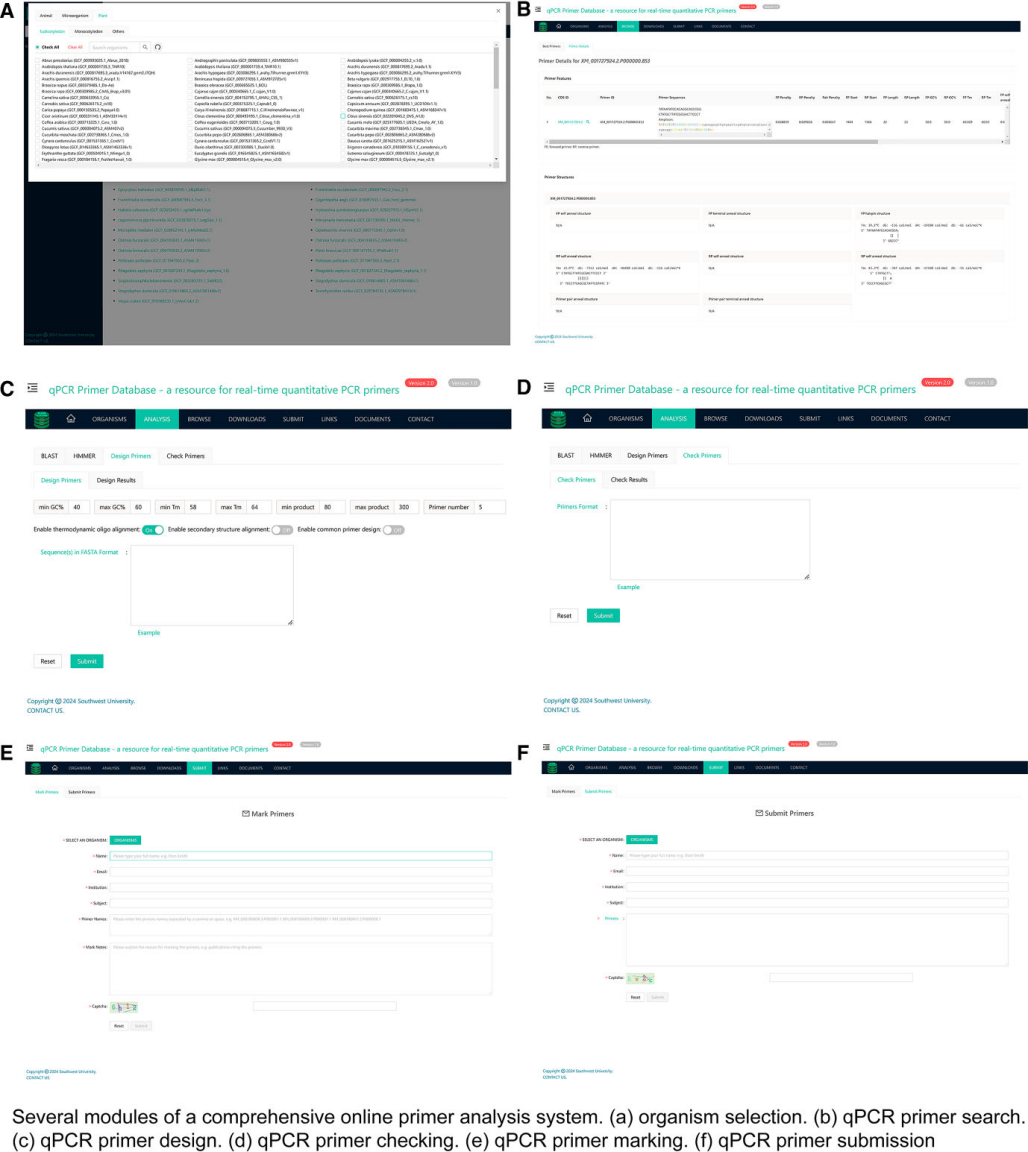
Several modules of a comprehensive online primer analysis system. (**A**) organism selection. (**B**) qPCR primer search. (**C**) qPCR primer design. (**D**) qPCR primer checking. (**E**) qPCR primer marking. (**F**) qPCR primer submission.

Best practices for the first step of selecting qPCR primers are to search for the target gene. qPrimerDB 2.0 provides gene-specific and pre-computed qPCR primer pairs from over 1 100 organisms for researchers to query (Figure 3A). The best primer design information can be accessed by entering the gene ID, gene symbol or primer ID directly. When primers are present in qPrimerDB 2.0, the search results will comprise two sections (Figure 3B): (i) basic primer features, including gene name, primer penalty score, GC content, and Tm value. Furthermore, several buttons are available for displaying amplicon details and primer alignments. Notably, each gene is associated with a link to the NCBI database. Additionally, exon-spanning information and the specificity of primer pairs will be included in primer alignments. (ii) Detailed primer secondary structure information, such as the propensity for hairpin and dimer formation.

#### Primer design

It is challenging to update primer resources into databases considering the rapid development of sequencing technology and iteration of genome versions. Therefore, we developed the ‘Design Primers’ feature in the ‘ANALYSIS’ module to bridge this gap and quickly provide high-quality primer design results for organisms of interest (Figure 3C). Considering that multiple variants of each gene may exist, the ‘Design Primers’ feature can also design common primers for multiple sequences. Therefore, best practices for the second step are to design new qPCR primers *de novo* that are not retrieved in qPrimerDB.

#### Primer check

The third step of the best practices is to check the quality of custom-designed primers for subsequent gene expression assays. The ‘ANALYSIS’ module offers a novel primer analysis feature called ‘Check Primers’, which assesses the quality of the primer design by computing basic primer features (Figure 3D). Furthermore, the ‘Check Primers’ feature also provides primer alignments and amplicon details upon selecting a reference sequence and inputting a template sequence.

#### Primer mark and submission

To enhance the reliability and reusability of primers, validated primers from published literature can be designated in the ‘MARK’ module and will be suggested first to experimental biologists when retrieving high-quality qPCR primers (Figure 3E). Additionally, the ‘SUBMIT’ module offers qPCR primer data submission services (Figure 3F). For convenience, users just need to provide the PMID and publication title; the qPCR primer information will be curated and incorporated into qPrimerDB 2.0 by expert curators. Detailed instructions for primer mark and submission are available in the ‘DOCUMENTS’ module, which is also the final step of the best practices.

## Conclusion and perspectives

qPrimerDB 2.0 is the most comprehensive primer database to date, with a web interface that supplies 430 million gene-specific and pre-computed qPCR primer pairs for over 1 100 important organisms [including humans, mice, zebrafish (*Danio rerio*), yeast (*Saccharomyces cerevisiae*), Arabidopsis, rice, and maize (*Zea mays*)]. The updated version provides a genome-scale primer design tool and numerous additional genomic resources. These features ensure continual updating of the database. A comprehensive primer analysis system will also allow for wider application of the database. We plan to include more PCR primer types, such as chromatin-immunoprecipitation (ChIP)-qPCR. Collectively, qPrimerDB 2.0 is a valuable, timesaving resource for gene expression analysis and will be updated routinely.

## Supplementary Material

gkae684_Supplemental_File

## Data Availability

qPrimerDB 2.0 is freely available online at https://qprimerdb.biodb.org. The qPrimer source code is available on GitHub repository at https://github.com/swu1019lab/qPrimer.
