# Supplementary material for: qPrimerDB 2.0: an updated comprehensive gene-specific qPCR primer database for 1172 organisms
Source: Nucleic Acids Res. 2024 Aug 9;53(D1):D205–10. doi: 10.1093/nar/gkae684 (PMC11701640; doi:10.1093/nar/gkae684)
Supplement: gkae684_Supplemental_File [file gkae684_supplemental_file.pdf]

a

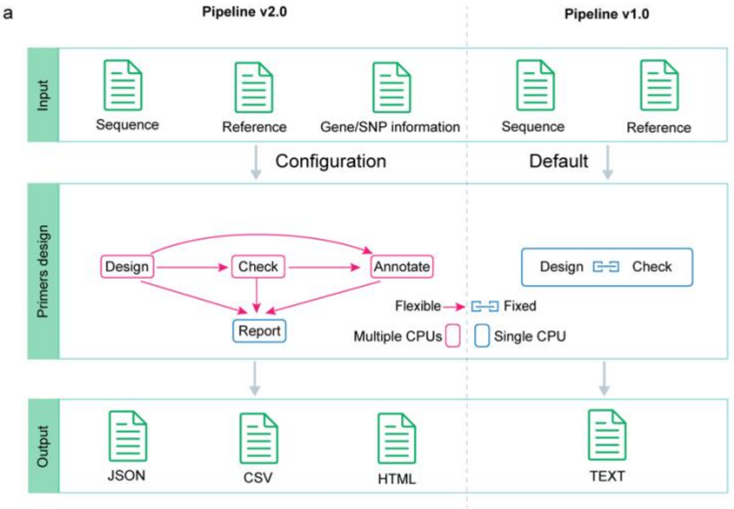

b

		Pipeline v2.0	Pipeline v1.0
Script type		Python	Shell
Input format		Fasta	Fasta
Function	Primer design	Available	Available
	Primer check	Available	Available
	Primer annotate	Available	NA
	Primer report	Available	NA
Multiple CPUs		Available	NA
Parameters config		Available	NA
JSON output		Available	NA

c

Organism	version	gene number	Pipeline 1.0		Pipeline 2.0	
			cover gene number	elapsed time (h)	cover gene number	elapsed time (h)
Arabidopsis thaliana	GCF_000001735.3_TAIR10.1	27,562	20,756	120.07	24,442	15.61
Oryza sativa	GCF_001433935.1_IRGSP-1.0	28,738	24,049	108.53	25,018	13.52
Zea mays	GCF_902167145.1_Zm-B73-REFERENCE-NAM-5.0	34,337	25,696	160.91	28,037	21.01

Supplementary Figure S1. The comparison and performance of pipeline between qPrimerDB v1.0 and v2.0. (a) and (b): The comparison of pipeline between two versions. (c) The performance of pipeline between two versions.

Supplementary Figure S1. The comparison and performance of pipeline between qPrimerDB v1.0 and v2.0. (a) and (b): The comparison of pipeline between two versions. (c) The performance of pipeline between two versions.
